# Thematic Reanalysis in the Left Posterior Parietal Sulcus: A TMS Study

**DOI:** 10.1162/nol_a_00043

**Published:** 2021-08-20

**Authors:** Chiara Finocchiaro, Luigi Cattaneo, Carlotta Lega, Gabriele Miceli

**Affiliations:** Department of Psychology and Cognitive Sciences, University of Trento, Trento, Italy; Center for Mind/Brain Sciences (CIMeC), University of Trento, Trento, Italy; Department of Psychology, University of Milan Bicocca, Milan, Italy; Beniamino Segre Interdisciplinary Center, National Academy of the Lincei, Rome, Italy

**Keywords:** transcranial magnetic stimulation, thematic role assignment, reanalysis, posterior parietal sulcus

## Abstract

Understanding *who does what to whom* is at the core of sentence comprehension. The actors that contribute to the verb meaning are labeled thematic roles. We used transcranial magnetic stimulation (TMS) to verify the possible impact of verb semantics on the thematic role encoding process that has been shown to involve the posterior portion of the left posterior parietal sulcus (PPS; Finocchiaro et al., 2015). Sixteen participants underwent TMS and sham stimulation sessions while performing an agent-decision task, in which they had to decide by key press which of the two arguments was the agent of visually presented sentences or pseudo-sentences. The (pseudo)sentences were all reversible and were presented in the active or passive diathesis. Double pulse TMS was delivered to the posterior part of the intraparietal sulcus in an event-related fashion, at two different time windows: 200–400 ms (T1) or 600–800 ms (T2) time-locked to the presentation of the (pseudo)sentence. Results showed that TMS increased accuracy on passive sentences and pseudo-sentences as compared to active sentences and to the baseline, sham condition. Indeed, the presence of a verb with a full semantic representation was not a necessary precondition for the TMS-induced facilitation of passive (pseudo)sentences. Stimulation timing had no effect on accuracy for sentences vs. pseudo-sentences. These observations support the idea that the posterior parietal site is recruited when the correct interpretation of a sentence requires reanalysis of temporarily encoded thematic roles (as in reversible passive sentences) even when the verb is not an entry in the lexicon and hence does not have a semantic representation. Results are consistent with previous evidence and deserve further investigation in larger experimental samples. Increasing the number and variety of stimulus sentences, and administering TMS to additional control sites will be key to further articulate the conclusions allowed by these initial findings.

## INTRODUCTION

The ability to understand *who does what to whom* is a core function of sentence comprehension. Indeed, understanding a verb means knowing the arguments it takes and the thematic roles it assigns to them (Chomsky, 1965, 1981). The *who does what to whom* structure contains all the main thematic roles: the agent is *who* does the action, the theme is *who/what* is involved in that action, the goal is the point to which that action is directed (*to whom*). Across languages, the most commonly used verbs are those whose thematic grid—in which the thematic roles associated to a given verb are specified—requires the roles of agent and theme. In Indo-European languages like English or Italian these verbs tend to correspond to transitive verbs (i.e., those that usually refer to an action performed by someone and involving someone/something else). When a transitive verb is used in the active diathesis, the thematic role of its arguments (agent and theme, respectively) matches their syntactic function (subject and object, respectively). For instance, in the sentence *Judith bites the apple*, Judith is both the subject of the sentence and the agent of the *bite*-action, and the apple is both the object of the sentence and the theme of the *bite*-action. Except for a few idiomatic expressions that are not admissible in the passive voice, transitive verbs normally allow the passive transformation. That is, the same event as *Judith bites the apple* can be described by the passive sentence *The apple is bitten by Judith*. The apple is now the subject of the sentence, but its thematic role is the same as in the active sentence (i.e., theme) as the apple does not start the *biting*-action but is still the element that undergoes biting. Judith is the agent because she gives origin to the biting, even though she is no longer the subject of the sentence and is now introduced by a *by-phrase*. Similar considerations apply to a sentence like *Judith bites Claire*. In the active diathesis, Claire is both the object and the theme but, when the sentence is transformed in the passive diathesis (*Claire is bitten by Judith*), Claire becomes the grammatical subject while remaining the logical theme.

There is, however, a major difference between the two sentences *Judith bites the apple* / *The apple is bitten by Judith*, and the two sentences *Judith bites Claire / Claire is bitten by Judith*: Whereas pragmatic cues are available for the first sentence pair, thus allowing one to assign the agent role to Judith irrespective of the sentence diathesis, the same cues are not available for the second sentence pair. This is because we know that apples do not bite people, whereas we do not have any expectations about the biting habits of women whose names are Judith or Claire. Therefore, sentences like *Judith bites Claire* and its passive version are called reversible (both arguments could in principle be agents); by contrast, sentences like *Judith bites the apple* and its passive version are called irreversible (the two arguments have a very different likelihood of being the agent).

Note that bare thematic violations do not violate argument structure, and as such are different from subcategorization violations in which the argument realization is not allowed by the verb (Garnsey et al., 1997; McElree & Griffith, 1995). Thus, in *The apple bites Judith*, there is no syntactic violation, but *the apple* is not marked for animacy as would be required for being the agent of a biting action. There is also a critical difference between active and passive sentences. In the passive diathesis there is a systematic mismatch between the expected order of the syntactic constituents and their thematic roles. In sentence comprehension, speakers show the tendency to interpret the first argument of a sentence as the subject and the agent of the denoted event (Ferreira, 2003; Grodzinsky, 2000; Meyer et al., 2012). This linear order cue may help the comprehension of active sentences, but leads to incorrect predictions for passive sentences, thus complicating the comprehension of passives and forcing the listener to reanalyze temporarily encoded thematic roles. Moreover, the passive diathesis is particularly difficult when, in addition to linear order cues being deceptive, the application of pragmatic cues also fails (i.e., in reversible passive sentences). Turner and Rommetveit (1967) proposed a hierarchy of sentence complexity, starting from the least complex nonreversible active, to reversible active, nonreversible passive, and ending with the most complex reversible passive.

Because of the absence of pragmatic or word order cues, reversible passives have been used to isolate the syntactic component of sentence comprehension in both neuropsychological and neuroimaging studies. In their seminal paper, Brookshire and Nicholas (1980) showed that aphasic and nonaphasic participants were slower and less accurate on passive than on active reversible sentences. The first report of a highly selective difficulty in the production and comprehension of thematic roles was provided by Caramazza and Miceli (1991). Patient EB, despite being able to flawlessly process the morphological structure of a sentence, was impaired in both comprehension and production of reversible sentences, especially (but, not exclusively) when sentences were passive. Other studies focused on reversible sentences as a means to manipulate the order (canonical vs. noncanonical) of the structural elements subject-verb-object (S-V-O). In Ferreira (2003), healthy participants were slower and less accurate in identifying the agent of reversible than nonreversible sentences, especially when the canonical S-V-O order was violated (e.g., reversible passives). Using the eye-tracking-while-listening paradigm, Meyer et al. (2012) found that upon hearing the first argument (N1) of active and passive sentences both aphasic and control participants immediately looked at the picture in which N1 was the agent.

A growing number of imaging studies have focused on the neural correlates of thematic role assignment. Studies reporting on comprehension disorders in aphasic patients, with emphasis on reversible sentences in the active and passive diathesis (Magnusdottír et al., 2013; Rogalsky et al., 2017; Thothathiri et al., 2012), documented substantial lesion overlap in posterior temporal and parietal regions (superior temporal, angular, and supramarginal gyri). In a study by Thompson et al. (2013) agrammatic patients were trained to exploit the argument properties of verbs through tasks that required verb naming and sentence generation. After training, the authors observed an improvement in verb production that generalized to untrained verbs and recruited posterior perisylvian regions as well as parietal and sensory-motor cortices bilaterally.

Studies also show that the complexity of a verb’s thematic grid can influence the neural mechanisms involved in the comprehension of reversible sentences. Thematic role complexity has been defined in different ways, as (1) the number of arguments a given verb may take (Thompson et al., 2010); (2) the transitive vs. intransitive opposition as a particular instance of the contrast between one-argument vs. two-argument verbs; (3) the number of thematic grids that may be associated with a single verb (Meltzer-Asscher et al., 2013). Indeed, alternating-transitivity verbs may be associated to more than one thematic grid, as they allow for transitive as well as intransitive use (e.g., *to eat* in *Judith has already eaten*, intransitive, and in *Judith has already eaten her sandwich*, transitive).

As to the neural correlates of verb comprehension processes, most fMRI studies show increasing activity in a (mostly left-sided) posterior perisylvian network and in the parietal lobe, that parallels increasing thematic role complexity. These phenomena involve the same regions found to be associated with verb production after training in Thompson et al. (2013).

Other neuroimaging studies used sentences instead of words. In Richardson et al. (2010), participants were asked to listen to or read silently reversible and nonreversible sentences. The left temporo-parietal boundary was found to be significantly more activated for reversible as opposed to nonreversible sentences. Mack et al. (2013) contrasted active and passive sentences and found greater activation for passives in the inferior frontal gyrus bilaterally and in left temporal regions.

The picture that emerges from the fMRI literature, despite differences across studies, converges with the neuropsychological literature in showing the involvement of left temporo-parietal regions in the assignment of thematic roles. In a recent study, Finocchiaro et al. (2015) used focal transcranial magnetic stimulation (TMS) to investigate the role of the parietal cortex in thematic role assignment. Focal TMS has an immediate effect on the neural activity of the stimulated portion of cortex, so that any TMS-related change in behavioral performance is likely to reflect the physiological function of the stimulated region. In their study, a reversible active or passive sentence was presented together with a picture. Participants had to judge, by key press, whether or not the sentence matched the picture (sentence-picture matching task). Three sites covering the whole length of the intraparietal sulcus (IPS) were stimulated with a short train of event-related double pulse TMS. Accuracy for reversible passive sentences increased following stimulation of the most posterior site along the IPS (probably corresponding to area V6A in the monkey) commonly thought to process visual information in a spatial frame of reference and possibly specialized in establishing relations between different components, as in depth judgments with objects or textures (Grefkes & Fink, 2005). Finocchiaro et al. interpreted their results as showing that the neural machinery in the posterior IPS is involved in linguistic processes and specifically in the assignment of thematic roles, especially when the reanalysis of transiently encoded thematic roles is required, as is the case in passive reversible sentences.

There is no consensus, however, on the theoretical status of the notion of thematic role. Fillmore’s original view (Fillmore, 1968) of thematic roles as primitive, verb-independent entities has been replaced by a number of accounts that vary greatly as to the participation of verb-specific semantics in the qualification of the agent and the theme. One of the most influential models was proposed by Dowty (1989, 1991). According to that model, thematic roles may be represented as groups of prototypical entailments imposed by a group of verbs on their arguments. Taken individually, the features associated to “proto-agents” and “proto-patients” are not necessary and sufficient to define an argument as agent or patient/theme, but together they contribute to the likelihood that a given argument is interpreted as an agent or a patient/theme. That is, whereas the “volitional involvement in the event or state” (Dowty, 1991, p. 572) is an entailment associated to protypical agents, there could be less prototypical agents that do not volitionally start the event/state. Along the same line, the model interprets other features more or less prototypically associated with agents or patients/themes. Other accounts, though not incompatible with Dowty’s view, emphasize the importance of verb-specific semantics for the characterization of thematic roles (McRae et al., 1997). These approaches focus on the characteristics normally associated with the thematic roles of agent and patient/theme of a specific verb. On this view, the interest shifts from verb-independent features to the verb-specific features associated with the agent of, for example, *to kill* as opposed to *to hide*, aiming at a richer characterization of the features that agents and patients may receive depending on the verb. (Other studies have stressed the importance of typological concepts such as prominence scales [animacy, definiteness/specificity, case marking, and linear order] on the comprehension of simple transitive sentences, [e.g., Bornkessel et al., 2005; Bornkessel-Schlesewsky & Schlesewsky, 2009]. These concepts are not directly addressed in our study.)

Be this as it may, all these accounts share the view that thematic roles cannot be assigned independently from verb semantics (see also Van Valin’s, 1999, notion of “generalized semantic roles”; for a review, see Lebani et al., 2015, and Levin & Rappaport Hovav, 2005). The goal of the present study is to better qualify the nature of the thematic role encoding processes shown to involve the posterior portion of the left parietal sulcus (PPS) in Finocchiaro et al. (2015). The crucial question is whether the semantics of a verb is crucial for the facilitatory effect on passives that follows PPS stimulation.

To answer this question, we presented participants with pseudo-sentences in addition to real sentences. If verb-specific semantic features are considered in the reanalysis process for which the PPS is recruited, the facilitation effect should be limited to real sentences. If, on the other hand, the process relies on primitive semantic features related to a proto-meaning of the type *someone did or suffered something*, facilitation should extend to pseudo-sentences, for which that proto-meaning may be recovered.

It is important to point out that “reanalysis” is used here as a theoretically-free label. In this study, we deliberately do not take a position in the linguistic debate on the involvement of syntactic movement in passive sentences. Our point is that both movement-based accounts (e.g., Chomsky, 1981) and lexical-thematic accounts (Bresnan, 2000; Pollard & Sag, 1994) share the view that passive sentences—as well as other types of noncanonical sentences—require a process of thematic reanalysis in order to revise the initial mapping of thematic roles. This process is not required in the case of active sentences (and canonical sentences in general).

Introducing pseudo-sentences led to a major difference in the experimental design. In Finocchiaro et al. (2015), TMS was delivered during a sentence-picture verification task, in which participants were asked to decide whether a written sentence was the appropriate description of the action represented in a picture. In the current study, pictures could not be used as stimuli, as pseudo-sentences cannot be depicted. Consequently, the sentence-picture matching task was substituted with an agent-decision task. In this task, the stimulus (pseudo)sentence is presented at the top center of the screen. The stimulus, being reversible, has two arguments. The two arguments are presented simultaneously below the (pseudo)sentence, one on top of the other. The participant is asked to decide by key-press which of the two arguments is the agent.

Based on the results reported in Finocchiaro et al. (2015), we focused on the stimulation site shown to be involved in the processing of passive sentences, that is, the posterior portion of the left IPS. The simplification of the overall TMS design allowed introducing a sham TMS condition as a within-participant variable, which permitted a quantitative as well as a qualitative comparison between the sham TMS and the TMS conditions.

We also introduced two different timings of TMS. In the present study, double pulse TMS was delivered at 200–400 ms or at 600–800 ms, in order to explore whether the effects of TMS varied as a function of the stimulation window and/or of the sentence/pseudo-sentence status of the target. Since sentence comprehension is an incremental process, showing that the effects of TMS differ as a function of stimulation timing may shed light on the time window in which reanalysis takes place.

To sum up, we expect to replicate previous findings by showing the involvement of the PPS in the processing of passive sentences relative to active sentences. In addition, manipulating the amount of semantic information available for verbs (complete for real sentences, very embryonal for pseudo-sentences) should allow inferences about the nature of the information that is relevant for the PPS. Since the PPS is not known to have a specific semantic role, we also expect the effect of TMS on passive processing to involve both sentences and pseudo-sentences. On the other hand, the manipulation of the TMS timing is exploratory, as we are not aware of relevant findings that could help shape our predictions.

## MATERIALS AND METHODS

### Participants

Sixteen native Italian speakers took part in the experiment for course credit. They were all right-handed (Oldfield, 1971), aged between 19 and 35 years (mean: 24.7), and had an educational level between 14 and 23 years (mean: 18). All participants had normal or corrected-to-normal vision, normal hearing, and no history of neurological or psychiatric disorders. They were preliminarily screened for any relative or absolute contraindication to TMS, received exhaustive information on the TMS procedure, and signed an informed consent form. Information about the purpose of the study was provided at the end of the test session. The study was approved by the ethics committee of the University of Trento.

### Materials

Two sets of stimuli were created. Stimuli in the first set (*N* = 60) were sentences containing real words; stimuli in the second set (*N* = 60) were pseudo-sentences containing pseudo-words. Pseudo-sentences contained real free-standing (determiners, prepositions, auxiliaries) and bound grammatical morphemes (gender and number morphemes for nouns; tense, mood, and person morphemes for verbs) but pseudo-word roots. Sentences and pseudo-sentences were presented half in the active and half in the passive diathesis, and they were all reversible (as the verb arguments used in pseudo-sentences are meaningless, there is no pragmatic constraint on their thematic roles).

Pseudo-words used in the pseudo-sentence set had an *N*-count (i.e., number of real words that can be obtained by substituting one letter at a time in each pseudo-word) equal to 0. Forty-three transitive verbs and 92 nouns were used in the sentence set. They appeared once or twice (in this latter case, in different blocks) during the experiment. Sentences and pseudo-sentences were of the same length in terms of number of (pseudo)words (5 for active (pseudo)sentences, 6 for passive (pseudo)sentences). We paid particular attention to excluding verbs that denoted spatial relations (e.g., follow, precede), as there is evidence that the processing of spatial relations may recruit parietal regions (Amorapanth et al., 2010; Baumann & Mattingley, 2014).

Stimuli were divided into three blocks of 40 items each. The four experimental conditions (sentence, active; sentence, passive; pseudo-sentence, active; pseudo-sentence, passive) were equally represented in each block. Sixteen additional stimuli were used as practice trials. They were presented to participants before the experiment proper began. They had the same characteristics as the experimental stimuli and were equally distributed across the experimental conditions.

### Procedure

**Figure 1.**
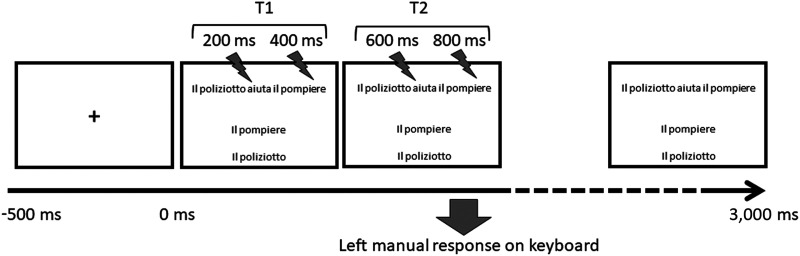
Schematization of trial timeline and structure.

The study was completed in one session that lasted about one hour per participant. The experiment was preceded by a practice block with the same characteristics as the experimental blocks, except that TMS was not delivered. The practice session could be repeated for a maximum of three times until the participant produced at least 80% correct responses. The experiment proper was repeated twice, once with TMS and once with a sham TMS. The order of presentation of the two conditions was counterbalanced across participants. For the TMS condition, double pulse TMS was delivered at T1 (between 200 and 400 ms) or T2 (between 600 and 800 ms). The same number of pulses was delivered at T1 and T2 for sentences and pseudo-sentences. The rationale for exploring these specific time periods with TMS is threefold. First, the average response time (RT) in this and similar tasks is around 1,500 ms. During this time window, the participant reads the sentence and the two alternative targets (see Figure 1) and produces a manual response. Given our experimental question, we aimed at exploring the initial and middle part of the RT, when the participant is actually reading the complete sentence. Second, studies on syntactic violations with event-related potentials hint that syntactic processing occurs in three specific time windows, starting at 150 ms up to 800 ms from the syntactic feature (Friederici, 1995, 2002; Friederici & Kotz, 2003). Third, active and passive sentences have syntactic features in different positions and are likely to be processed at slightly different timings, hence the need to explore at least two separate time windows.

At the beginning of each trial a fixation cross appeared on-screen for 500 ms, immediately followed by the stimulus. Each stimulus consisted of a full (pseudo)sentence center aligned in the upper half of the screen (e.g., *Il poliziotto aiuta il pompiere* ‘The policeman helps the fireman’ or *Il pompiere è aiutato dal poliziotto* ‘The fireman is helped by the policeman’), which appeared at the same time as the two arguments (*Il pompiere* ‘The fireman’ and *Il poliziotto* ‘The policeman’), shown one above the other, center aligned in the lower half of the screen. The position of the two arguments was counterbalanced across conditions, so that the agent was above or below the theme an equal number of times. The stimulus remained on-screen until participants responded or 3,000 ms had elapsed, whichever came first. Participants were asked to read the sentence and to decide, by pressing the “B” or the “N” key with their left hand, whether the agent was the argument appearing in the top or in the bottom line. The agent was previously defined as the person who does the action denoted by the verb. We chose the left hand as effector to avoid any interference of left hemispheric TMS on the motor processes involved in producing a hand movement. A single trial is schematically represented in Figure 1. Overall, the study was designed for repeated measures with trials fully distributed according to four within-subject factors: TMS (effective or sham), TIME of stimulation (two levels), SEMANTICS (word or pseudo-word), DIATHESIS (active or passive). The presentation of the stimuli within each block was completely randomized. The order of block presentation was counterbalanced across participants according to a Latin-square design. Stimulus presentation was controlled by E-Prime software (https://pstnet.com/products/e-prime/).

### TMS

**Figure 2.**
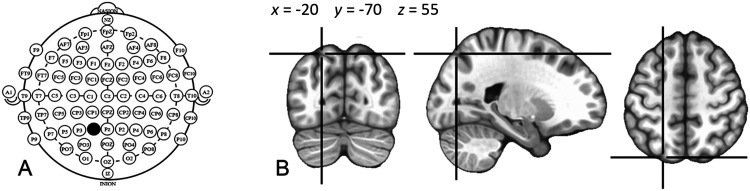
(A) Reference coordinates of the international 10-20 system for EEG electrode placement. Coordinate P1, used in the present study as stimulation target is indicated in black. (B) According to probabilistic scalp-brain atlases (Herwig et al., 2003; Kabdebon et al., 2014; Koessler et al., 2009; Okamoto et al., 2004; Sparing et al., 2010; Yoo et al., 1997), the cortical projection of the P1 scalp coordinate is probabilistically distributed *between-subjects* over a region that has a maximum of probability (i.e., is localized in most subjects) at the −20, −70, 55 coordinates of the standardized MNI space, indicated in the figure with the crosshair on a standard MNI brain.

Magnetic stimuli were delivered with a double 70 mm figure-eight coil connected to a Magstim Rapid (The Magstim Company, UK) biphasic stimulator that was externally triggered by TTL pulses at appropriate timing by the E-Prime software through the parallel port of the computer. Following up on the results obtained by Finocchiaro et al. (2015), we delivered the double pulse TMS to the posterior third of the left IPS (i.e., to the site where TMS affected the processing of passive sentences). In the present study, the TMS target was identified by means of the international 10-20 coordinates (see Figure 2). Several atlases provide probabilistic maps of the cortical structures that correspond to specific 10-20 system coordinates on the scalp (Herwig et al., 2003; Kabdebon et al., 2014; Koessler et al., 2009; Okamoto et al., 2004; Sparing et al., 2010). Based on these atlases we identified the P1 coordinate (located halfway between the Pz and P3 coordinates in the extended 10-20 system) as the one most probably overlying the posterior part of the IPS. The coil was oriented so as to be roughly perpendicular to the IPS, at 90° to the midline, the handle pointing medially. Stimulation intensity was individually set to 100% of the visually-assessed motor threshold (i.e., to the intensity required to produce a visible twitch in hand muscles in exactly 50% of eight trials). The sham TMS condition was achieved by tilting the coil by 90°, so that only the edge of the TMS coil touched the scalp and therefore the subject experienced the mechanical and acoustical experience of effective TMS, without actual cortical stimulation.

### Data Pre-processing

Analyses were carried out on RTs and on accuracy (Acc). The experimental design was based on repeated measures with four within-subject factors: TMS (2 levels: sham or effective), DIATHESIS (2 levels: active or passive), SEMANTICS (2 levels: sentence or pseudo-sentence) and TIME (2 levels: TMS delivered at 200–400 ms [T1] and TMS delivered at 600–800 ms [T2]). There were 15 trials in each cell of this 2 × 2 × 2 × 2 design. Trials were trimmed according to RTs. Any trials with RTs longer or shorter than the mean +/−2 standard deviations (*SD*s) were excluded from further analysis. As expected from a normal distribution, RTs fell >2 *SD* below the mean in 1% of the trials. Additionally, we excluded from further analysis trials in which no response was given after 3 s. After data trimming, the numerosity of trials/cell went from 15 to an average of 14.1, ranging from 10 to 15. Trials with incorrect responses were excluded from the RT analysis, but were obviously included in the Acc analysis.

### Statistical Analysis

Linear mixed-effect models were used (Baayen et al., 2008). The model was estimated using R (R Development Core Team, 2016) and the *lme4* package (Bates et al., 2015). Post hoc tests were conducted using the R-package *phia* (version 0.2-0; De Rosario-Martinez et al., 2015) and applying the Bonferroni-Holm correction for multiple comparisons. A mixed logistic model was estimated using accuracy as the dependent variable. TMS, DIATHESIS, SEMANTICS, and TIME and their interaction were entered as fixed-effect factors in a mixed logistic model that predicted accuracy. Intercepts were also included in the model as random-effect factors across participants and across session. An identical analysis was performed on RTs as the dependent variable.

### Effect Sizes

Calculating effect size for mixed models is difficult, and there is little consensus among statisticians on how to do it (see Nakagawa & Schielzeth, 2013). Reporting the effect size for analyses such as mixed-effects regression modeling and hierarchical linear modeling can be difficult. For these reasons, most reports so far have avoided reporting effect size when using mixed model analysis. One of the best approximations of effect size for general linear model is Cohen’s *f* ^2^ (Selya et al., 2012), “which allows an evaluation of local effect size, i.e., one variable’s effect size within the context of a multivariate regression model” (Selya et al., 2012, p. 1). Cohen’s *f* ^2^ is calculated starting from the R^2^, which is the proportion of variance accounted for by the model. Mixed models have 2 different types of R^2^: the marginal R^2^, which represents the variance explained by the fixed effects, and the conditional R^2^, which represents the variance explained by the entire model, including both fixed and random effects. Therefore, we calculated two different *f* ^2^ for each significant effect, namely the *f*_m_^2^ (marginal) and the *f*_c_^2^ (conditional). Notably, for multi-level models, effect sizes calculated using residual variance and proportion of variance explained should be interpreted with caution, because adding variables to the model may increase residual variance resulting in negative estimates of explained variance and even of effect size (Snijders & Bosker, 1994). In the present work we proceeded as follows. The model was estimated using R (R Development Core Team, 2016) and the *lme4* package (Bates et al., 2015). Post hoc tests were conducted using the R-package *phia* (version 0.2-0; De Rosario-Martinez et al., 2015) and applying the Bonferroni-Holm correction for multiple comparisons. A mixed logistic model was estimated using accuracy as the dependent variable. TMS, DIATHESIS, SEMANTICS, and TIME and their interaction were entered as fixed-effect factors in a mixed logistic model that predicted accuracy. Intercepts were also included in the model as random-effect factors across participants and across session.

## RESULTS

### Planned Analysis

None of the participants reported side effects, immediate or delayed. Accuracy values and latencies are presented in Tables 1 and 2.

**Table 1.** Accuracy values (%) in all experimental conditions

TMS type	TMS time	Active diathesis		Passive diathesis	
		Word	Pseudo-word	Word	Pseudo-word
Effective TMS	t1	89.2 (3.2)	91.2 (2.3)	90.9 (3.0)	93.3 (2.5)
	t2	86.4 (5.0)	87.1 (3.8)	88.8 (4.8)	88.0 (2.8)
Sham TMS	t1	89.7 (3.3)	93.4 (2.5)	84.6 (4.2)	84.5 (4.0)
	t2	88.8 (3.7)	88.8 (2.4)	81.1 (4.9)	87.3 (3.3)

*Note*. The standard error of the mean is indicated in parentheses.

**Table 2.** Reaction times (ms) in all experimental conditions

TMS type	TMS time	Active diathesis		Passive diathesis	
		Word	Pseudo-word	Word	Pseudo-word
Effective TMS	t1	1,670 (74)	1,696 (73)	1,764 (79)	1,836 (78)
	t2	1,678 (79)	1,797 (76)	1,756 (64)	1,740 (72)
Sham TMS	t1	1,677 (67)	1,748 (80)	1,756 (71)	1,773 (71)
	t2	1,724 (71)	1,798 (58)	1,751 (73)	1,757 (78)

*Note*. The standard error of the mean is indicated in parentheses.

**Figure 3.**
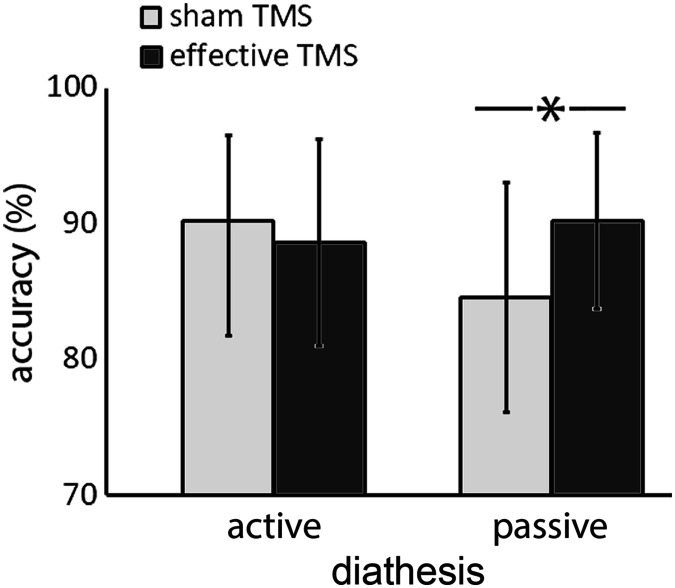
TMS and DIATHESIS interaction. Asterisk denotes significant difference. Error bars represent standard deviation of the mean.

The analysis on RTs did not yield any effect of interest (i.e., any effect including the TMS factor). Conversely, the analysis on accuracies revealed a significant main effect of DIATHESIS, (*Chi* ^2^ (1) = 4.18, *p* = 0.040, *f*_m_^2^ = 0.001, *f*_c_^2^ = 0.002), and a main effect of TIME (*Chi* ^2^ (1) = 7.28, *p* = 0.006, *f*_m_^2^ = 0.002, *f*_c_^2^ = 0.003). The interaction between TIME and DIATHESIS was also significant (*Chi* ^2^ (1) = 11.33, *p* < 0.001, *f*_m_^2^ = 0.003, *f*_c_^2^ = 0.003) (see Figure 3). Post hoc analyses (Bonferroni-Holm correction) indicated a significant difference between sham and effective TMS for passive DIATHESIS (*Chi* ^2^ (1) = 12.58, *p* < 0.001), but not for active DIATHESIS (*Chi* ^2^ (1) = 2.07, *p* = 0.14). None of the other main effects or interactions reached significance (all *p*s > 0.08).

## DISCUSSION

In a recent TMS study, Finocchiaro et al. (2015) showed increased comprehension accuracy for passive sentences relative to active sentences following stimulation of the left posterior IPS. The present study replicates and extends those findings, as the same facilitation for passives was obtained when directly contrasting within-participant performance under sham and TMS conditions in an agent-decision task. In line with Finocchiaro et al. (2015), we interpret this result as showing that the posterior IPS is involved in thematic role assignment, specifically when transiently encoded thematic roles need to be reanalyzed.

The main goal of the present paper was to check whether the semantics of the verb is crucial for the facilitation of passive diathesis interpretation. On one hand, the most credited linguistic accounts posit the involvement of verb semantics in thematic role encoding (Dowty, 1989, 1991; Lebani et al., 2015; Levin & Rappaport Hovav, 2005; McRae et al., 1997; Van Valin, 1999). On the other hand, the PPS is not known to be devoted to semantic processing, and thus the type of information relevant to the PPS may not concern verb-specific semantics. To investigate this issue, both sentences and pseudo-sentences were presented. Simple reversible sentences in active and passive diathesis were randomly alternated with active and passive pseudo-sentences, which contained real free-standing and bound grammatical markers but pseudo-word roots. Critically, both passive sentences and passive pseudo-sentences were facilitated, and the magnitude of the effect was comparable for the two stimulus types. It should be noted that the main contrast on which our results are based is the comparison between the effects of effective TMS applied to one single spot with the effects of sham TMS. In the present work we focus on the posterior portion of the IPS, which was shown to be involved in diathesis processing in Finocchiaro et al. (2015). The results reported here clearly show that the meaningfulness of the verb is not a necessary precondition for the repetitive TMS induced facilitation of passive sentences. Perhaps the nature of the pseudo-sentences (pseudo-word roots, real grammatical morphemes) allowed participants to recover a proto-meaning of the stimulus, according to which *someone did something to somebody else* or *someone suffered something because of somebody else*. In the absence of a full-verb’s semantics, participants may have treated the meaningless arguments as “proto-agent” and “proto-patient” respectively, thus attributing to them the features that are prototypically assigned to agents and patients/themes (Dowty 1989, 1991). In this framework, one can tentatively conclude that the process of reanalysis, at least to the extent to which it recruits the PPS, is relatively insensitive to verb-specific semantics (see McRae et al., 1997). This does not imply that the semantics of the stimuli has no influence whatsoever on reanalysis; rather, it suggests that the left posterior IPS is insensitive to the semantic characteristics of the stimuli that are relevant to the reanalysis process.

Remarkably, the TMS-induced facilitation for passives was not affected by stimulation timing. Indeed, performance did not significantly change depending on whether double pulse TMS was delivered at T1 (200–400 ms) or T2 (600–800 ms). It is commonly assumed that the integration of the lexical, semantic, and syntactic elements of a simple sentence is completed between 500 and 1,000 ms (Friederici, 2002). In the present experiment, stimulation time was manipulated in order to explore the incremental processing of active and passive (pseudo)sentences as it relates to the involvement of the left posterior IPS. Our results do not shed light on this issue, probably for reasons related to the complexity of the experimental task. Indeed, to complete each trial the participant had to read the sentence, analyze it, decide which argument was the agent, and finalize their choice. The duration of each step may vary across participants and even from trial to trial, thus leading to the huge variation in response latencies observed across and within participants. Such variability may have hidden differential effects (if any) of the stimulation timing.

The polarity of the effect deserves a separate comment. As in Finocchiaro et al. (2015), in the present experiment TMS improved accuracy on passive sentences. Finocchiaro et al. (2015) accounted for the TMS-induced facilitation on passives by assuming that “the posterior intraparietal site processes the relevant information after the TMS train is delivered” (page 230). In light of the lack of effects of different TMS timings on performance, this explanation is unlikely. It has been assumed that the neural and behavioral effects of TMS depend on the state of the cortex being stimulated (Pasley et al., 2009; Perini et al., 2012; Silvanto et al., 2008). The TMS of neurons with lower-than-average firing patterns is more likely to facilitate behavior, whereas the TMS of neurons with hyper-active firing patterns is more likely to disrupt it. This model has been repeatedly supported in the literature in experimental designs in which the excitability of a given neural population is manipulated by adaptation or priming procedures (Barchiesi et al., 2012; Cattaneo, 2010; Cattaneo et al., 2010, 2011; Mazzoni et al., 2017; Silvanto et al., 2007). On this account, the polarity of TMS effects is unpredictable unless the initial cortex excitability is known. Since the present experiment was not designed to highlight state-dependent effects, no clear conclusion can be drawn on this issue.

Our data show that TMS-induced facilitation for passive sentences and pseudo-sentences is not tied to full-verb semantics, and that it is probably linked to the fact that interpreting passive sentences requires the reanalysis of thematic roles. This effect could be interpreted purely in terms of working memory. Indeed, many studies have shown that the posterior parietal region is involved in the encoding of thematic roles as well as in working memory processes in sentences presented both auditorily and visually (Papagno et al., 2007; Romero Lauro et al., 2006; Romero Lauro et al., 2010). The data also show that area BA40 is strictly tied to the phonological loop (Romero Lauro et al., 2010). The task used in the experiment discussed here requires participants to keep phonological information active by relying on their phonological store (Baldo & Dronkers, 2006). However, an account based entirely on working memory cannot accommodate our results. On the reasonable assumption that working memory demands are greater when processing pseudo-sentences, which are more difficult to keep in memory than real sentences, such an account would predict a difference between passive sentences and passive pseudo-sentences, with pseudo-sentences showing a greater facilitation effect.

Another potential account of the observed effects could stem from consideration of the key role of the posterior portion of the parietal lobe in encoding spatial relations (Amorapanth et al., 2010; Baumann & Mattingley, 2014). It has been proposed that thematic relations can be translated into non-linguistic spatial representations (Chatterjee et al., 1995; Coslett, 1999). On this view, the effect putatively attributed to thematic reanalysis could be reduced to a spatial effect. At face value, this possibility is unlikely, as we expressly avoided using verbs or prepositions that denote relative positions or trajectory (e.g., *precede*, *follow*, *in front of*, *behind*, *above*, *below*). Moreover, the argument hardly applies to pseudo-sentences, which do not convey any obvious spatial content.

Overall, an account of the role of left PPS in sentence processing in terms of thematic role encoding fits better with evidence from neuropsychological (Thothathiri et al., 2012) and fMRI studies (Mack et al., 2013; Meltzer-Asscher et al., 2013; Richardson et al., 2010; Thompson et al., 2010, 2013) showing the involvement of temporo-parietal regions in the processing of thematic roles, particularly for reversible and passive sentences. For example, a correlation between impaired performance on reversible sentences and temporo-parietal damage was observed in aphasic participants by Magnusdottír et al. (2013), Rogalsky et al. (2017) and Thothathiri et al. (2012). Moreover, Thothathiri et al. (2012) also showed that difficulty with thematic roles persists after controlling for canonical/noncanonical word order and covarying for working memory. In combination with these data, the present results add information on the dynamics of the sentence comprehension process, via a technique that allows a fine-grained temporal resolution during an agent-decision task.

We applied TMS to the posterior third of the left IPS. This region is referred to as superior parietal-occipital cortex in humans and is considered homologous to areas V6A and PEc in nonhuman primates. The posterior portion of IPS has been dubbed as the “parietal reach area,” based on its key role in behavioral tasks (Battaglia-Mayer et al., 2014; Galletti & Fattori, 2018; Grefkes & Fink, 2005). It is embedded in the “dorsal” visual stream (Ungerleider & Mishkin, 1982), which encodes visual stimuli in distinct but mostly body-centered frames of reference. Like all dorsal stream regions, it is assumed to encode visual information for goal-directed action and transform it into targets of reaching movements. Owing to the integration of visual and somatosensory information, it also presumably provides online feedback on reaching movements (Gallivan & Culham, 2015; Grefkes & Fink, 2005; Fattori et al., 2012; Piserchia et al., 2017). Consistent with this view, classical neuropsychology reports show that damage to the posterior portion of the IPS and the superior parietal lobule results in optic ataxia (e.g., Andersen et al., 2014), that is, the inability to direct reaching movements to a half of a visual space. Given the anatomic-functional aspects of posterior IPS, an intriguing question arises: Why should an area unanimously deemed critical for visuospatial behavior be relevant for the processing of passive sentences?

The anatomical localization of the TMS target is not entirely precise. We refer to it as the posterior part of the IPS, but obviously TMS cannot reach the deeper part of its banks; therefore, the current data suffer from an anatomical bias toward the surface of the IPS region. This said, the posterior-superior portion of IPS has been shown to be involved in nonmotor tasks, such as the processing of series of digits or letters (Bueti and Walsh, 2009; Eger et al., 2003; Jacob & Nieder, 2008; Marshuetz et al., 2001; Schubotz et al., 2000; Schwenzer & Mathiak, 2011). A core feature of all these tasks is the need to encode the serial order of the elements comprising the stimulus. Crucially, encoding the order of single elements is also a critical feature of sentence processing. Thus, the effect reported here may be linked to the processing of argument order, or more likely reflect the mapping from argument order to thematic roles, systematically reversed in passive sentences.

In this framework, the hypothesis that a cortical region that implements spatial representations also plays a critical role in syntactic processing is worth considering. Syntax relies strongly on order. Scrambling word order relations in a sentence affects syntax, but not other language functions. The human brain is well known to use its machinery flexibly, for apparently heterogeneous functions. Based on the view that the brain can successfully accomplish the “cultural recycling of cortical maps” (Dehaene & Cohen, 2007, p. 384), behavioral requirements that are relatively recent could be supported by neural hardware originally evolved for different functions. Other cognitive domains in the human behavioral repertoire have been linked to networks dedicated to other functions in nonhuman beings, as is the case of reading (Dehaene & Cohen, 2011), time perception, or action understanding (Cattaneo, 2010; Schubotz et al., 2000). The same may have happened for mechanisms involved in aspects of syntactic processing that pose similar computational demands in nonlanguage contexts.

### Limitations

Even though the findings are promising and in line with a previous TMS study, some methodological shortcomings suggest caution in the interpretation of the results and must be overcome in future work. A first issue is related to the lack of a control site. Given that the main contrast is between TMS and sham TMS when applied to one single spot, it cannot be completely ruled out that the observed TMS effects are due to general arousal or alerting effects. In fact, sham TMS, even though conducted according to conventional experimental practice, does not provide participants with the full sensory experience of effective TMS. Indeed, applying TMS to just one cortical spot without any active TMS control sites may lead to circular reasoning in which the premises of the experiment turn out to be the conclusions (Cattaneo, 2018). This possibility is partially mitigated by the consideration that the present experiment capitalizes on the results of previous work in which the entire linear extension of the IPS had been mapped with 3 active TMS spots, showing that passive sentences were affected only by stimulation of the spot selected for the present experiment (Finocchiaro et al., 2015). Still, the presence of a control site would strengthen the reliability of the data reported here. Other concerns stem from the limited sample size (16 participants) and the small number of trials in each experimental condition. Indeed, for each stimulation type (effective vs. sham) three variables were considered: semantics, diathesis, and time (i.e., stimulation timing T1 vs. T2), each with two levels, thus leading to only 15 trials per participant in each experimental condition. The low number of participants may undermine the possibility of generalizing the observed effect to different participants and items and therefore suggests caution. In fact, percentage scores in the region of 80–90% correspond to changes in accuracy of only 1 or 2 items per condition. Further studies with more participants, an increased number of trials in each experimental condition, and an additional control site should assess the reliability of the present findings.

## ACKNOWLEDGMENTS

The authors wish to thank Martina Costamagna for her precious support in data collection.

## FUNDING INFORMATION

The study was funded in part by a grant from PAT (Provincia Autonoma di Trento) to GM.

## AUTHOR CONTRIBUTIONS

**Chiara Finocchiaro**: Conceptualization: Equal; Resources: Equal; Writing – original draft: Lead; Writing – review and editing: Lead; Supervision: Lead. **Luigi Cattaneo**: Conceptualization: Equal; Formal analysis: Lead; Resources: Lead; Writing – original draft: Equal; Writing – review and editing: Lead. **Carlotta Lega**: Formal analysis: Lead; Resources: Equal; Writing – review and editing: Supporting. **Gabriele Miceli**: Conceptualization: Equal; Funding Acquisition: Lead; Writing – original draft: Equal; Writing – review and editing: Supporting; Supervision: Equal. The first two authors (Chiara Finocchiaro and Luigi Cattaneo) contributed to this project to an equal extent and thus share the role of first author.

## TECHNICAL TERMS

Thematic role: Semantic role that a given noun phrase may play in relation to the action/event/state denoted by a governing verb.
Reversible sentences: Sentences for which both arguments could in principle be agents (there is no semantic cue).
Pseudo-sentences: Sentences made up of pseudo-words. Word-endings and function words are real elements in a given language.
Pseudo-words: In a given language, possible but not existent strings of graphemes/phonemes.
